# Metallothionein ameliorates airway epithelial apoptosis upon particulate matter exposure: role of oxidative stress and ion homeostasis

**DOI:** 10.1007/s44194-024-00036-7

**Published:** 2024-09-10

**Authors:** Bin Li, Nannan Huang, Shengnan Wei, Qingtao Meng, Shenshen Wu, Michael Aschner, Xiaobo Li, Rui Chen

**Affiliations:** 1 School of Public Health, Capital Medical University, Beijing 100069, China.; 2 Key Laboratory of Environmental Medicine Engineering, Ministry of Education, School of Public Health, Southeast University, Nanjing, China.; 3 Department of Molecular Pharmacology, Albert Einstein College of Medicine, Forchheimer 209, 1300 Morris Park Avenue, Bronx, NY 10461, USA.; 4 Advanced Innovation Center for Human Brain Protection, Capital Medical University, Beijing 100069, China.; 5 Institute for Chemical Carcinogenesis, Guangzhou Medical University, Guangzhou 511436, China.; 6 School of Public Health, Southeast University, Nanjing 210009, China.

**Keywords:** Particulate matter, Oxidative stress, Ion homeostasis, Metallothionein

## Abstract

**Purpose:**

To investigate the mechanism underlying particulate matter (PM) exposure-induced oxidative stress and potential rescue strategies against pulmonary damage in this context.

**Methods:**

A combination of omics technology and bioinformatic analysis were used to uncover mechanisms underlying cellular responses to PM exposure in human bronchial epithelia (HBE) cells and imply the potential rescue.

**Results:**

Our results implicated that oxidative stress, metal ion homeostasis, and apoptosis were the major cellular responses to PM exposure in HBE cells. PM exposure disrupted oxidative phosphorylation (OXPHOS)-related gene expressions in HBE cells. Rescuing the expression of these genes with supplemental coenzyme Q10 (Co Q10) inhibited reactive oxygen species (ROS) generation; however, it only partially protected HBEs against PM exposure-induced apoptosis. Further, metallothionein (MT)-encoding genes associated with metal ion homeostasis were significantly induced in HBE cells, which was transcriptionally regulated by specificity protein 1 (SP1). SP1 knock-down (KD) aggravated PM-induced apoptosis in HBE cells, suggesting it plays a role in MT induction. Subsequent studies corroborated the protective role of MT by showing that exogenous MT supplement demonstrated effective protection against PM-induced oxidative stress and apoptosis in HBE cells. Importantly, exogenous MT supplement was shown to reduce ROS generation and apoptosis in airway epithelia in both HBE cells and a PM-inhaled murine model.

**Conclusion:**

This study demonstrates that the impact of MT on airway epithelia by suppressing oxidative stress and maintaining metal ion homeostasis is beneficial in attenuating damage to pulmonary cells undergoing PM exposure.

## 1. Introduction

Urban particulate matter (PM) in ambient air is a heterogeneous mixture of particle sizes and chemicals. PM typically originates from mobile and industrial fossil fuel combustion, and its specific constituents and sources have not been fully elucidated. The adverse health effects of PM exposure have been associated with respiratory-related morbidity and mortality (Li et al. 2018; Zhang 2021; Bowe et al. 2019). Airway inflammation, systemic inflammation, and oxidative stress have been implicated in experimental animals or longitudinal panel studies (Feng et al. 2023; Xu et al. 2022).

The respiratory track epithelial cells are at the interface between the airspace and the internal milieu, acting as a physical and biochemical barrier to ensure transition between these distinct compartments. Airway epithelia cells can be activated by a host of stimuli, including air pollutants (Fu et al. 2019; Barbier et al. 2023). In the context of activation, epithelial cells provide an adaptive response to these stimuli, which is important for the elimination and wound healing, and the generation of a systemic response (Crotty Alexander et al. 2018; Gangwar et al. 2020). Therefore, airway epithelia are the first line of defense against air pollutants, as well as the primary target of inhaled PM in organisms.

Numerous studies have focused on the mechanisms underlying PM exposure-induced adverse health effects. Overall, these studies corroborate that oxidative stress mediates, at least in part, PM-induced pulmonary toxicity (Gangwar et al. 2020; Lan et al. 2021). Nonetheless, gaps in knowledge delineating the precise role of ROS in air pollution-mediated pathologies have yet to be fully characterized (Gangwar et al. 2020). Furthermore, with consistent and inevitable air pollution exposure, additional studies investigating novel efficient therapeutic interventions are urgently required (Whyand et al. 2018).

In the present study, human bronchial epithelial cells (HBE) and mice were exposed to various concentrations and doses, respectively, of PM. Omics technology and biological assays were used to explore the molecular pathways underlying pulmonary toxicity of PM. The cellular source of ROS generation and underlying mechanisms upon PM exposure was delineated. Importantly, our findings identified a putative rescue strategy against PM-induced toxicity, shedding novel insight on intervention strategies and the putative role of MT in rescue from PM-induced pulmonary damage.

## 2 Material and methods

### 2.1 Particulate matter (PM)

Urban Particulate Matter (Standard Reference Material 1648a) were purchased from National Institute of Standards and Technology, USA. The components of SRM 1648a were introduced by a research group from NIST (Schantz et al. 2012).

### 2.2 Cell culture

The human bronchial epithelial cells (HBE, American Type Culture Collection) were maintained in Dulbecco’s modified Eagle’s medium (DMED) at 37 °C in 5% CO_2_. The culture medium was supplemented with 10% (v/v) fetal bovine serum (FBS), penicillin (100 U/mL), streptomycin (100 μg/mL).

### 2.3 Cellular viability

Cellular viability was evaluated with a Cell Counting Kit-8 (CCK-8, Nanjing Jiancheng Bioengineering Institute, China). According to the reconciliation between in vitro and in vivo doses of PM (Li et al. 2003), PM-induced biological effects at the dose range of 0.2–20 μg/cm2 in vitro were equal to that at the dose of 75 μg/m3 over a 24-h period which is PM_2.5_ limit recommended in China. HBE cells were plated at a density of 1 × 104 per well in a 96-well plate and then exposed to 100 μL PM mixture (0, 12.5, 25, 50, 100, 250, 500, and 1, 000 μg/mL) with three biological replicates for each concentration, equal to PM (0, 0.16, 0.33, 0.65, 1.3, 3.25, 6.5, 13 μg/cm2) for 24 h. Cell viability affected by PM were monitored every 24 h up to 3 days. Then 10 μL CCK-8 solution was added to each well, the cells were incubated for 4 h at 37 °C, and the absorbance was determined at 450 nm.

### 2.4 RNA microarray and gene expression analysis

HBE cells were seeded in 10 cm culture dishes and exposed to 0, 100, and 500 μg/mL PM, respectively, with three biological replicates. Total cellular RNA was extracted after 24 h treatment with the TRIZOL reagent (Invitrogen, US), according to the manufacturer’s instructions (Agilent Technologies, Santa Clara, CA, US), and subjected to microarray assay. The labeled cRNAs were hybridized onto a Human LncRNA Array v3.0 (8 × 60 K; Arraystar) chip, which is designed for 26, 109 coding genes. The arrays were scanned with an Agilent G2505C scanner and the density of fluorescence was analyzed with Agilent Feature Extraction software (version 11.0.1.1). Quantile normalization and subsequent data processing were performed with GeneSpringGx v12.0 software package (Agilent Technologies). An absolute fold change (FC) of 1.5 or more and *p* = 0.05 were set as threshold to evaluate the significance of gene expression differences of raw data.

### 2.5 Functional group analysis

The DAVID 6.7 (Database for Annotation, Visualization and Integrated Discovery, https://david.ncifcrf.gov/) functional annotation tool was used to denote the significance of gene ontology (GO) term enrichment in the differentially expressed mRNAs (*p*-value was set less than 0.1) based on a background of homo sapiens genome.

### 2.6 Animal treatments

Male C57BL/6 mice (20–22 g), were purchased from GemPharmatech Co. Ltd. (China) and housed in a specific pathogen-free (SPF) animal facility, in which the room temperature was maintained at 22.5 °C with 12 h light/dark cycle. Mice were housed six per polycarbonate cage on corncob bedding with ad libitum access to food and water. Mice received standard rodent chow (Jiangsu Xietong Pharmaceutical Bio-engineering Co., Ltd., China) sterilized by cobalt (Co) 60 radiation and autoclaved water ad libitum. Animals were treated humanely, maintained, and used in accordance with Guidelines of Committee on Animal Use and Care of Southeast University (20190226014).

Mice were divided into six groups (*n* = 6/group), namely filtered air (FA)/Vehicle, PM (300 μg/m3)/Vehicle, PM (600 μg/m3)/Vehicle, FA/metallothionein (MT), PM (300 μg/m3)/MT, and PM (600 μg/m3)/MT. Exposure was carried out as previously described (Li et al. 2021; Meng et al. 2022). In brief, mice were placed in three stainlesssteel Hinners-type whole-body inhalation chambers; two chambers were ventilated with PM, and the remaining one with HEPA-filtered clean air at the same flow rate as the PM exposure chamber. Mice were exposed for 2 h per day for 30 consecutive days. MT were administrated at a dose of 2.5 mg/kg, i.p., 1 h before PM exposure every 3 days. Control mice received equal volume of 0.9% saline injection.

Mice were deeply euthanized under isoflurane anesthesia 1 h after the end of PM exposure on the 30th day. A portion of the left lung tissue from each mouse was fixed in 4% paraformaldehyde (PFA), and the remainder of the lung tissues were stored in liquid nitrogen.

### 2.7 RNA isolation and quantitative real‑time PCR assay

HBE cells were exposed to 0, 50, 100, 250, or 500 μg/mL PM for 24 h. Next, cells were trypsinized and collected. Total RNA was extracted with the GenEluteTM Mammalian Total RNA Miniprep Kit (Sigma, US). cDNA synthesis for coding genes was performed with 1 μg of total RNA according to the manufacturer’s instruction (Toyobo, Tokyo, Japan).

The mRNA levels for target genes were determined by reverse transcription of total RNA followed by quantitative real-time PCR analysis (qRT-PCR) on a Quant Studio 6 Flex system (Applied Biosystems, Life Technologies, US) with SYBR PCR Master Mix reagent kits (Toyobo, Japan). Primer sequences are shown in Table S1. All experiments were independently performed in triplicate. The mRNA levels for the indicated gene are relative to cyclophilin A (CycA).

### 2.8 JC‑1 staining

Changes in mitochondrial membrane potential (Δψm) in pulmonary cells were determined with a commercial 5,50′,6,6′-tetrachloro-1,1′,3,3′-tetraethyl-benzimidazolyl-carbocyanine iodide (JC-1) kit (C2003S, Beyotime, China). HBE cells were exposed to PM for 24 h, added with JC-1 staining working solution, and incubated at 37 °C for 20 min. After the incubation, HBE cells were washed twice with JC-1 staining buffer and evaluated under a fluorescence microscope (Olympus, Japan) to examine green and red fluorescence, the relative fluorescent intensities were determined by the ratio of green/red fluorescent intensity with Photoshop software.

### 2.9 ROS generation

ROS levels in HBE cells and murine lung tissues were determined with a commercial ROS assay kit (S0033S, Beyotime Biotechnology, China). After exposure to 0, 50, 100, 250, or 500 μg/mL PM for 24 h, HBE cells were washed with PBS. Then 2’, 7’-dichlorofluorescein-diacetate (DCFH-DA) probe was added at a final concentration of 10 μmol/L and incubated at 37 °C for 30 min to determine the content of intracellular ROS.

Paraffin-fixed lung tissue sections were incubated with DCFH-DA at 37 °C for 20 min. The sections were covered with a mounting solution containing DAPI (DAPI Fluoromount-G, SouthernBiotech, USA), and then observed under a fluorescence microscope (Axio Imager M2, Zeiss, Germany). Three non-overlapping fields containing at least one small airway at low-power field (100×) of each section were randomly selected, the relative fluorescent intensities were determined by the ratio of green/DAPI fluorescent intensity with Photoshop software.

### 2.10 ATP levels

ATP levels were measured with a luciferase ATP assay kit (Beyotime, China). HBE cells were exposed to 0, 50, 100, 250, or 500 μg/mL PM for 24 h and added with 200 μL of lysis buffer. Cells were collected and centrifuged at 12,000 rpm for 5 min at 4 °C. The luminescence of the supernatant was assayed with a luminometer (Berthold Detection System, Pforzheim, Germany).

### 2.11 Cytochrome c contents

After exposure to 0, 50, 100, 250, or 500 μg/mL PM for 24 h, HBE cells were washed with PBS. Mitochondrial and cytosolic proteins of HBE cells were isolated by a Mitochondria/Cytosol Fractionation Kit (Beyotime, China) and subsequently quantitated using the Bradford method. The levels of cytochrome c were estimated according to the ELISA kit procedures (R&D Systems, U.S.).

### 2.12 Cell apoptosis analysis

PM exposure-induced cellular apoptosis was analyzed by flow cytometry with an Annexin V-FITC Apoptosis Detection Kit (KeyGEN BioTECH, China). Briefly, after exposure to 0, 50, 100, 250, or 500 μg/mL PM for 24 h, HBE cells were harvested and washed twice with PBS, followed by centrifugation at 1, 000 rpm for 5 min. Then the pellets were resuspended in 500 μL binding buffer and incubated with 5 μL FITC-conjugated annexin V and 5 μL PI for 15 min at room temperature in the dark. The samples were analyzed by FACS Calibur Flow Cytometry (BD Biosciences, US).

### 2.13 ELISA assays

HBE cells were exposed to 0, 50, 100, 250, or 500 μg/mL PM for 24 h. Protein content was measured by the Bradford method. HBE cells were incubated with Ac-DEVD-pNA, Ac-IETD-pNA, Ac-LEHD-pNA, which are substrate peptides of caspase-3, 8, and 9, respectively (Beyotime Institute of Bio-technology, China) for 2 h at 37 °C. Release of p-nitroanilide (pNA) from substrates was measured at 405 nm by a microplate reader (Molecular Devices, U.S.)

### 2.14 ChIP assay

ChIP was performed using the ChIP-IT^™^ Express Magnetic assay kit (Active Motif, USA). HBE cells were exposed to 0, 100, or 500 μg/mL PM for 24 h, and fixed with 4% formaldehyde for 10 min. The samples were then centrifuged at 2, 000 g for 2 min and washed once with cold PBS. The ChIP reaction antibody was a normal rabbit IgG (NI01, EMD Chemicals, Inc., Gibbstown, NJ) and an anti-SP1 (1:500 dilution; ab231778, Abcam, USA). Precipitated genomic DNA was analyzed by quantitative PCR in triplicate measurements for each sample using appropriate primers (Table S2).

### 2.15 Western blot

HBE cells were exposed to 0, 100, or 500 μg/mL PM for 24 h, and total proteins were extracted by a total protein extraction kit (Life Technologies, USA) from cells. A total of 20 μg of protein was subjected to electrophoresis. Blots were then incubated with primary antibodies against SP1 (1: 1000 dilution; ab231778, Abcam, USA) for 48 h, follow by incubation with HRP-conjugated secondary antibodies for 1 h. α-tubulin (1:5000 dilution; ab7291, Abcam, USA) was used as a loading control. The immunoreactive signals were visualized with a Super ECL Plus Kit (Yeasen, China).

### 2.16 TUNEL staining

Apoptotic cells in lung tissues were evaluated with terminal-deoxynucleoitidyl transferase mediated nick end labeling (TUNEL) staining by a Roche In Situ Cell Death Detection Kit (Roche, U.S.). The nuclear stained areas (depicted in dark brown) were identified as TUNEL-positive cell. The proportion of TUNEL-positive cells of an airway were estimated by two experienced histologists who were blinded to experimental design. Three to five non-overlapping airways in each section were counted in high-power fields (HPFS, × 400 magnification) and analyzed. The airways with the maximum number of positive cells were selected for analysis (Li et al. 2017).

### 2.17 Short interfering RNA (siRNA) construction and cell transfection

To knockdown the expression of SP1 and MT1F, SP1- or MT1F- targeting siRNA and scrambled negative control (NC) sequences were designed and synthesized by GenePharma (Shanghai, China). HBE cells were seeded in six-well plates and transfected with siRNAs using Lipofectamine 2000 Transfection Reagent (Invitrogen, USA) combined with Opti-MEM (Gibco, USA) in line with the manufacturer’s protocol. Forty-eight hours after transfection, knockdown efficiency was verified by qRT-PCR or WB. The sequences of siRNAs are listed in Table S3.

### 2.18 Coenzyme Q10 (Co Q10) supplementation

HBE cells were treated with Co Q10 (2.5 or 5.0 μM, C9538, Merck, Germany) for 6 h, then exposed to 0, 100, or 500 μg/mL PM for 24 h. Co Q10 was dissolved in ethanol as a stock solution. The stock solution was incubated at 37 °C for 15 min prior to addition to the HBE cells. Following evaluation of gene expression levels, a single dose of 5.0 μM Co Q10 supplement was used for ROS generation, caspase-3, −8, −9 ELISA assay, and apoptosis detections in HBE cells. The control group were supplemented with an equal volume of ethanol as in the 5.0 μM Co Q10 group.

### 2.19 MT supplementation

HBE cells were treated with 1μg/mL MT (rabbit liver, ALX-202–070, ENZO, USA)) dissolved in 0.9% saline for 6 h (Nygaard et al. 2015; Leung et al. 2018), then exposed to 0, 100, or 500 μg/mL PM for 24 h. Gene expression levels, ROS generation, caspase-3, −8, −9 ELISA assay, and apoptosis were evaluated in HBE cells as described above.

### 2.20 Histopathological analysis

Lung tissues were fixed in PFA for 24 h at 4 °C, embedded in paraffin, serially sectioned (5 μm) and mounted on silane-covered slides. The sections selected from each mouse were stained after dewaxing with hematoxylin and eosin (H&E) and evaluated under a light microscope (400 ×) to examine tissue histology. Images were scanned with the slide scanner Panoramic SCAN (3DHISTECH, Hungary) to obtain a whole slide image. Histology of murine lung tissues were evaluated by an experienced histologist who was blinded to experimental design.

### 2.21 Statistical analysis

Data are shown as means ± SD, unless indicated otherwise. For the comparison among multiple groups, one-way or two-way analysis of variance (ANOVA) followed with Dunnett’s multiple comparisons was used, as indicated in the figure legends. The results of qRT-PCR were analyzed by 2^−ΔΔCt^ method. Statistical significance was set at *p* < 0.05.

## 3 Results

### 3.1 mRNA expression profiles in HBE cells were altered by PM exposure

**Fig. 1:**
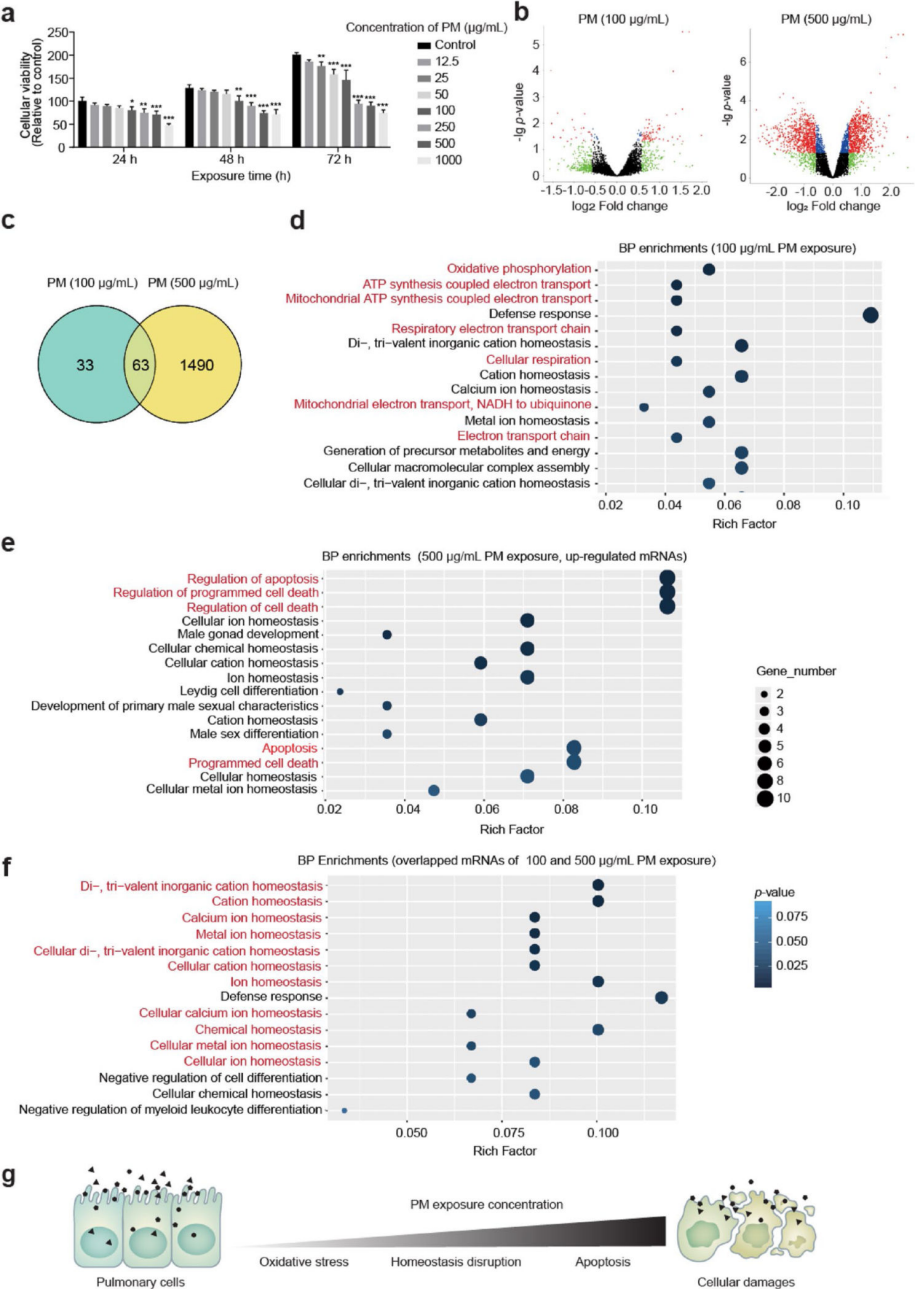
PM exposure alters mRNA expression profiles in HBE cells. **a** HBE cellular viability was inhibited in a concentration-depended manner following PM exposure. (*n* = 3/group, one-way ANOVA for each time point). **b** Volcano plots showed the differentially expressed genes following 100 or 500 μg/mL PM exposure for 24 h with a cut-off as fold change (FC) < 1.5 and *p*-value > 0.05 (*n* = 3/group). **c** Venn diagram showed the differentially expressed genes in 100 or 500 μg/mL PM-exposed HBE cells. **d** and **e** GO terms (biological function, BP) of differential expressed genes of 100 or 500 μg/mL PM-exposed HBE cells. **f** GO terms (BP) of differential expressed genes overlapped in 100 and 500 μg/mL PM-exposed HBE cells. **g** Schematic of PM exposure resulted cellular damages in HBE cells

The cellular viability of HBE cells was inhibited in a concentration-dependent manner following 24, 48, or 72 h PM exposure. The significant decrease in cellular viability (80.21 ± 7.63%, 74.62 ± 8.82%, 70.90 ± 7.61%, and 47.65 ± 3.56%, relative to the control) occurred in HBE cells following 100, 250, 500, and 1000 μg/mL PM exposure for 24 h, respectively (Fig. 1a). Two doses (100 and 500 μg/mL) of PM exposure, which resulted in cellular viability reduction of 20% and 30%, respectively, relative to the control, were chosen for the mRNA microarray assays. A total of 140 and 758 up-regulated genes, and 42 and 927 down-regulated genes were by 100 or 500 μg/mL in PM-exposed HBE cells for 24 h, respectively, with a cut-off of > 1.5-fold change (FC) and *p*-value < 0.05 (Fig. 1b). Venn diagram demonstrated the number of differentially expressed genes in 100 and 500 μg/mL PM-exposed groups (Fig. 1c). Gene ontology (GO) of differentially expressed genes suggested multiple enrichments related to mitochondrial oxidative phosphorylation in the 100 μg/mL PM-exposed HBE cells (Fig. 1d and Table S4); and apoptosis in 500 μg/mL PM-exposed HBE cells (Fig. 1e and Table S5). As for overlapped genes between 100 and 500 μg/mL PM-exposed groups (including 63 differentially expressed genes), they were highly enriched in ion homeostasis-related terms (Fig. 1f and Table S6). Therefore, we hypothesized that PM exposure exerted effects on HBE cells in a concentration-dependent manner. At the relatively low concentration, PM exposure triggered oxidative stress; with increased concentration, ion homeostasis was disrupted, eventually resulting in apoptosis in HBE cells (Fig. 1g).

### 3.2 PM exposure triggered oxidative stress by targeting phosphorylation (OXPHOS) in HBE cells

**Fig. 2:**
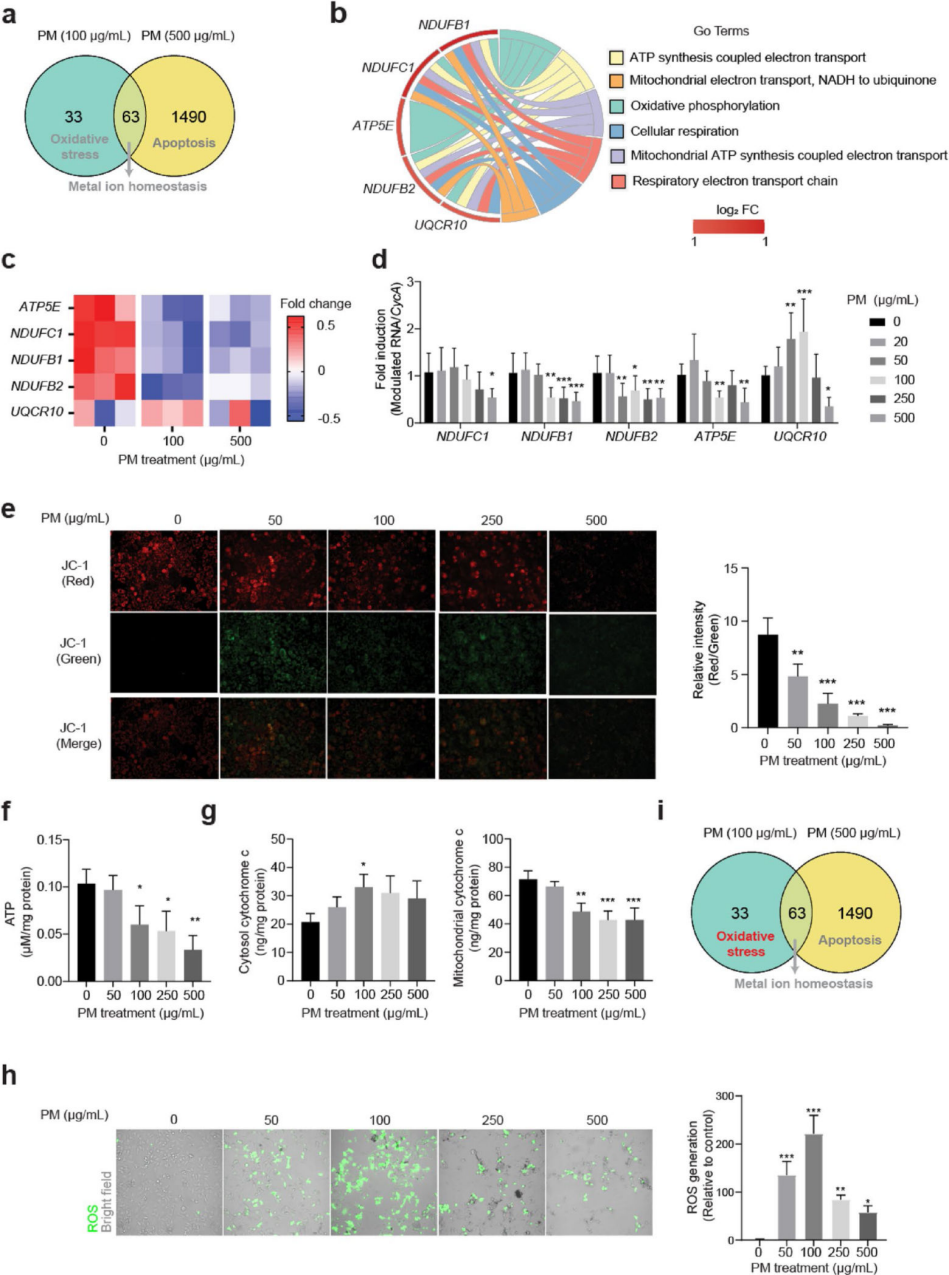
Low-concentration of PM exposure induces oxidative stress in HBE cells. **a** Venn diagram showed the differentially expressed genes and potential cellular responses to PM exposure in HBE cells. **b** Chord diagram of mitochondria-related GO terms and involved genes. **c** Heatmap of mitochondria-related genes in HBE cells following PM exposure for 24 h by microarray. **d** qRT-PCR assays of mitochondria-related genes in HBE cells following PM exposure for 24 h (*n* = 9/group, one-way ANOVA). **e** JC-1 staining of HBE cells following PM exposure for 24 h (*n* = 3/group, one-way ANOVA). **f** ATP production in HBE cells following PM exposure for 24 h (*n* = 3/group, one-way ANOVA). **g** Contents of cytosol and mitochondrial cytochrome c in HBE cells following PM exposure for 24 h (*n* = 3/group, one-way ANOVA). **h** ROS generation in HBE cells following PM exposure for 24 h (*n* = 3/group, one-way ANOVA). **i** Schematic of the cellular responses to low-dose PM exposure in HBE cells. * *p* < 0.05, ** *p* < 0.01, *** *p* < 0.001, compared to the PM (0 μg/mL) group

To confirm our hypothesis that relatively low concentration of PM triggered oxidative stress in HBE cells (Fig. 2a), the expression levels of 5 genes involved in mitochondrial function and OXPHOS (Fig. 2b and c) were validated. Consistent to the microarray data (Fig. 2c), expression levels of *NDUFC1*, *NDUFB1*, *NDUFB2*, and *ATP5E* were inhibited following 24 h PM exposure, and the expression levels of *UQCR10* were significantly increased at 50 and 100 μg/mL groups, while decreased at 500 μg/mL group (Fig. 2d) compared to the control. Mitochondrial functions were consistently disrupted by showing significant collapse of the mitochondrial membrane potential (Fig. 2e), decreased ATP content (Fig. 2f), increased cytosol cytochrome c levels, and decreased mitochondrial cytochrome c levels (Fig. 2g) in HBE cells following PM exposure relative to the control. PM exposure also significantly induced ROS levels in HBE cells. Among all the groups, the maximal ROS generation was observed in the 100 μg/mL PM-treated group (Fig. 2h). Therefore, it was confirmed that HBE cells responds to relatively low PM exposure by induction of oxidative stress (Fig. 2i).

### 3.3 PM exposure resulted in apoptosis in HBE cells

**Fig. 3:**
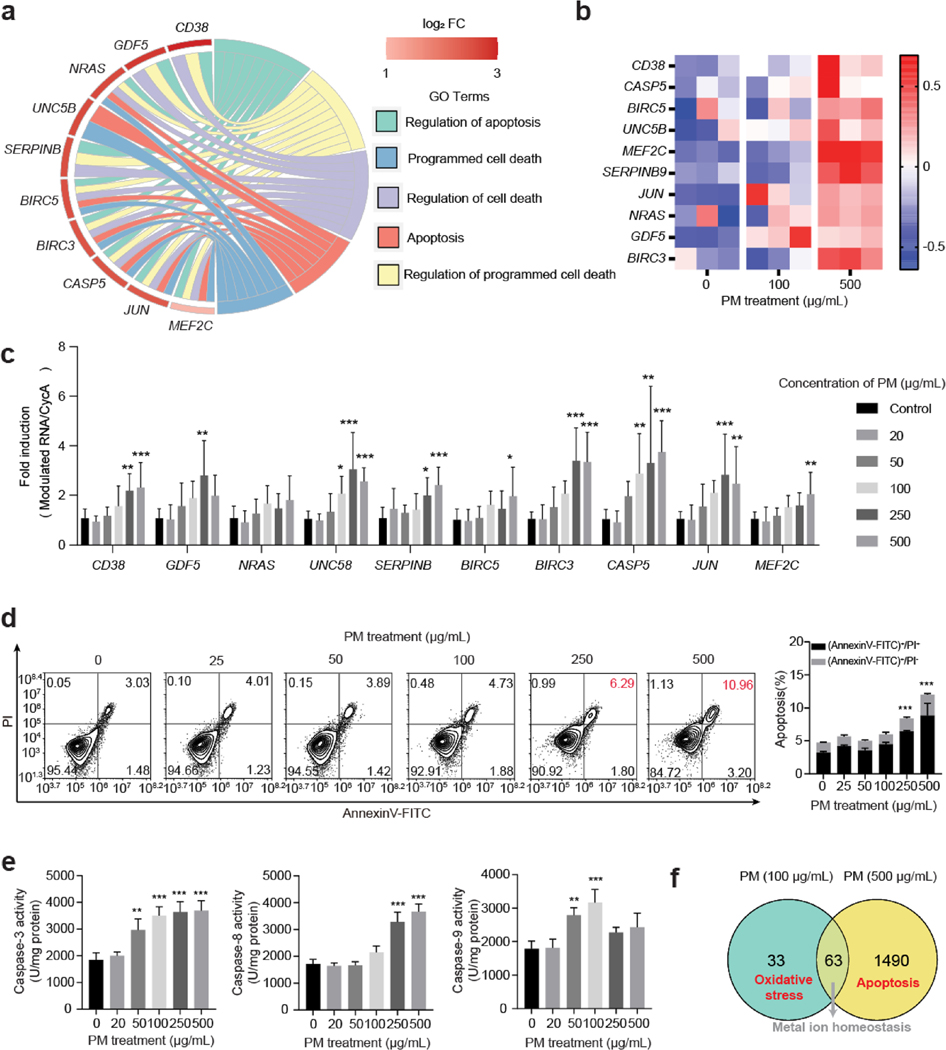
High-concentration PM exposure leads to apoptosis in HBE cells. **a** Chord diagram of apoptotic GO terms and involved genes in 500 μg/mL PM-exposed HBE cells. **b** Heatmap of apoptosis-related genes in HBE cells following PM exposure for 24 h. **c** qRT-PCR assays of apoptosis-related gene expression levels in HBE cells following PM exposure for 24 h (*n* = 9/group, one-way ANOVA). **d** Cellular apoptosis induced by PM exposure in HBE cells (*n* = 3/group, one-way ANOVA). **e** Levels of caspase-3, −8, 9 in HBE cells following PM exposure (*n* = 3/group, one-way ANOVA). **f** Schematic of the cellular responses to high-dose PM exposure in HBE cells. * *p* < 0.05, ** *p* < 0.01, *** *p* < 0.001, compared to the PM (0 μg/mL) group

To further confirm our hypothesis that high concentration of PM exposure resulted in apoptosis in HBE cells (Fig. 3a), the expression levels of apoptosis-related genes were validated by qRT-PCR assays. Consistent to the microarray data (Fig. 3b), 8 out of 10 apoptosis-involved genes were significantly induced in HBE cells following 24 h 500 μg/mL PM exposure (Fig. 3c). The flowcytometric assays showed significantly increased apoptosis in 250 and 500 μg/mL PM-exposed HBE cells for 24 h, relative to the control (Fig. 3d). According to previous study, Caspase-related apoptosis can proceed in intrinsic or extrinsic pathways (Shalini et al. 2015). In intrinsic apoptosis pathway, death-inducing stimulus, such as ROS and DNA damage, lead to activation of apoptosome following Caspase-9 cleavage and Caspase-3 activation. As for extrinsic apoptosis, it depends on complex formation by death-domain-containing proteins which activated Caspase-8 leading to apoptosis. In Fig. 3e, we identified the activation of mitochondria-dependent (Caspase-9) and non-mitochondria-dependent (Caspase-8) apoptosis pathways in HBE cells following low-concentration (50 and 100 μg/mL) and high-concentration (250 and 500 μg/mL) PM exposure respectively. Our results indicated two different pathways of PM-induced apoptosis in a dose-dependent manner. These results suggested that relatively high dose of PM exposure damaged HBE cells by inducing apoptosis (Fig. 3f).

### 3.4 MT‑encoding genes consistently responded to PM exposure in HBE cells

**Fig. 4:**
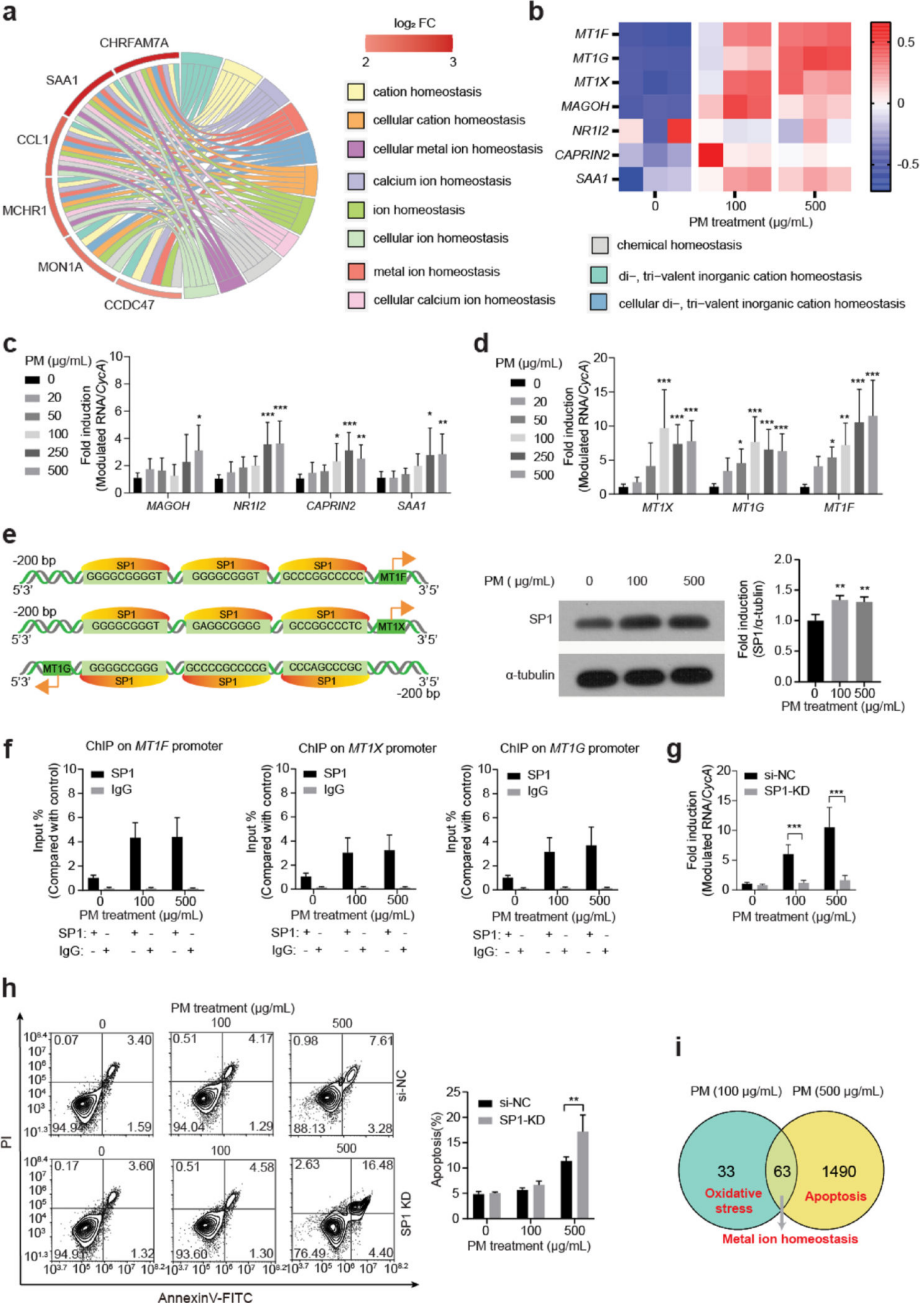
Disruption of ion homeostasis contributes to apoptosis in HBE cells following PM exposure. **a** Chord diagram of ion homeostasis GO terms and involved genes in 500 μg/mL PM-exposed HBE cells. **b** Heatmap of ion homeostasis-related genes in HBE cells following PM exposure for 24 h by microarray. **c** qRT-PCR assays of ion homeostasis-related genes in HBE cells following PM exposure for 24 h (*n* = 9/group, one-way ANOVA). **d** qRT-PCR assays of MT-encoding genes in HBE cells following PM exposure for 24 h (*n* = 9/group, one-way ANOVA). **e** Schematic of transcription factor SP1 binding to the promoter regions of MT-encoding genes and Expression levels of SP1 in PM-exposed HBE cells for 24 h (*n* = 3/group, one-way ANOVA). **f** ChIP assays verified that SP1 binding promoted MT1X, MT1F, and MT1G transcription ((*n* = 6/group, *t*-test). **g** SP1 knockdown inhibited expression of MT1F (*n* = 9/group, one-way ANOVA). **h** Apoptosis of HBE cells (control or SP1 KD) following PM exposure (*n* = 3/group, two-way ANOVA). **i** Schematic of the cellular responses to PM exposure in HBE cells. * *p* < 0.05, ** *p* < 0.01, *** *p* < 0.001, compared to the PM (0 μg/mL) group

Bioinformatic analysis showed that ion homeostasis-involved enrichments played a vital role in response to PM exposure in HBE cells (Fig. 4a). Among the 63 overlapped differentially expressed genes between 100 and 500 PM-exposed groups, a total of 7 genes were significantly induced with ≥ twofold change compared to the control (Fig. 4b). Four of the 7 genes, including *MAGOH*, *NR1I2*, *CAPRIN2*, and *SAA1*, were ion homeostasis GO enrichments-involved genes and their expression levels were significantly higher in PM-exposed groups compared to the control (Fig. 4c). The remainder 3 genes, including *MT1F*, *MT1G*, and *MT1X*, were MT-encoding genes.

MT is a well-known antioxidant and its transcriptions can be rapidly induced by stimuli, such as oxidative stress and exogenous metals exposure (Andrews 2000). Expression levels of MT-encoding genes were induced in HBE cells following PM exposure (Fig. 4d). Next, we investigated their up-stream drivers. SP1 was predicted to be a transcription factor binding to the promoter region of *MT1F*, *MT1X*, and *MT1G* and its expression levels were induced in HBE cells following PM exposure (Fig. 4e). ChIP assays showed that binding of SP1 to the promoter regions of *MT1F*, *MT1X*, and *MT1G* was enhanced in the PM-exposed group compared to the control (Fig. 4f).

Whether increased expression levels of MT-encoding genes in HBE cells following PM exposure have beneficial or deleterious role has yet to be determined. We constructed SP1 knock-down (KD) HBE cells and the expression of SP1 were ablated in HBE cells following PM exposure (Fig. S1a and Fig. 4g) significantly attenuated the induction of *MT1F*, *MT1X*, and *MT1G* expression levels in HBE cells following PM exposure (Fig. S1b); whereas, expression levels of MAGOH, NR1I2, CAPRIN2 were not transcriptionally regulated by SP1 in HBE cells following both 100 and 500 μg/mL PM exposure; expression levels of CAPRIN2 and SAA1 were significantly decreased in SP1-KD group only following 500 μg/mL PM exposure. (Fig. S1c). Furthermore, SP1 KD enhanced PM exposure-induced apoptosis in HBE cells compared to the control (Fig. 4h). As shown in Fig. 4d, the expression levels of *MT1F* were increased in a dose-dependent manner following PM exposure, we thus chose to knock down the expression of *MT1F* (Fig. S1d) in HBE cells as an example to assess whether MT-encoding genes act a protect role on PM-induced apoptosis. Indeed, we observed high apoptosis level in HBE cells following 24 h 100 and 500 μg/mL PM exposure in *MT1F* KD group (Fig. S1e). Therefore, the induction of MT-encoding genes was protective merely based on the increase of PM-induced apoptosis caused by SP1 KD. These results corroborated our hypothesis that with the increased PM exposure levels, oxidative stress, ion homeostasis disruption, and apoptosis were sequentially induced in HBE cells (Fig. 4i).

### 3.5 MT effectively protected HBE cells against PM exposure‑induced cellular damages

**Fig. 5:**
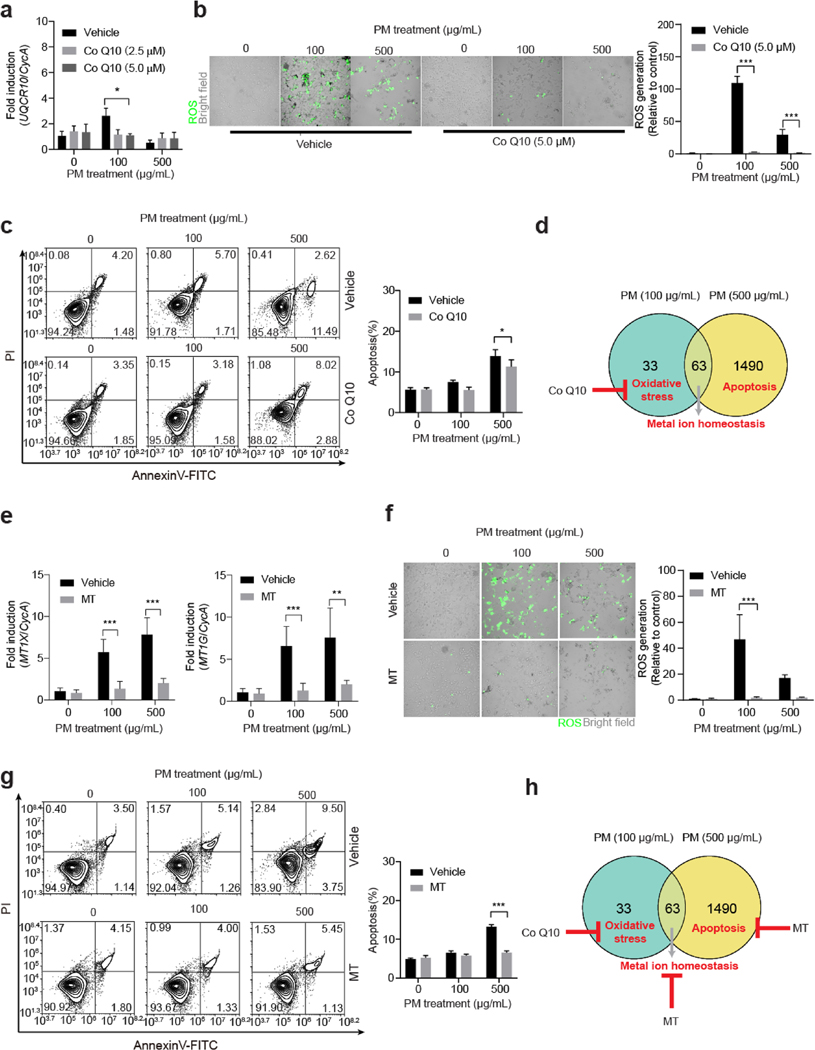
MT efficiently protects HBE cells against PM exposure-induced apoptosis. **a** qRT-PCR assays of UQCR10 expression in HBE cells following PM exposure with or without Co Q10 supplement (*n* = 9/group, two-way ANOVA). **b** ROS generation in HBE cells following PM exposure with or without Co Q10 supplement (*n* = 3/group, two-way ANOVA). **c** Apoptosis n HBE cells following PM exposure with or without Co Q10 supplement (*n* = 3/group, two-way ANOVA). **d** Schematic of the Co Q10 rescue against PM exposure-induced oxidative stress in HBE cells. **e** qRT-PCR assays of MT1X and MT1G expression in HBE cells following PM exposure with or without MT supplement (*n* = 9/group, two-way ANOVA). **f** ROS generation in HBE cells following PM exposure with or without MT supplement (*n* = 3/group, two-way ANOVA). **g** Apoptosis n HBE cells following PM exposure with or without MT supplement (*n* = 3/group, two-way ANOVA). **h** Schematic of the MT rescue against PM exposure-induced apoptosis in HBE cells. * *p* < 0.05, ** *p* < 0.01, *** *p* < 0.001, compared to the PM (0 μg/mL) group

We further explored the potential rescue to PM exposure-induced damage in HBE cells. CoQ10 is an electron carrier in the mitochondrial respiratory chain, an antioxidant, and anti-apoptotic factor (Emma et al. 2016). Here, Co Q10 supplement (5.0 μM) rescued the expression of UQCR10 to control levels in HBE cells following 100 μg/mL PM exposure (Fig. 5a), but failed to rescue the expression of *NDUFB1*, *NDUFB2*, *NDUFC1*, and *ATP5E* (Fig. S2). Co Q10 supplement significantly reduced ROS generation in 100 and 500 μg/mL PM-exposed cells (Fig. 5b). However, Co Q10 supplement only partially inhibited the levels of Caspase-3 and −9 in HBE cells (Fig. S3), and slightly attenuated apoptosis in 500 μg/mL PM-exposed HBE cells (Fig. 5c). Thus, though Co Q10 effectively inhibited ROS generation, it was not an ideal rescue reagent against PM exposure induced apoptosis in HBE cells (Fig. 5d).

Since induction of MT-encoding genes effectively inhibited PM exposure-induced apoptosis, we tested the effects of MT supplement on HBE cells. Following exogeneous MT supplement, the expressions of *MT1F*, *MT1X*, and *MT1G* in PM-exposed HBE cells were statistically indistinguishable from control levels (Fig. 5e and Fig. S4a). The expressions of mitochondria-related genes were not affected by MT supplement (Fig. S4b to f). MT displayed anti-oxidative efficacy by inhibiting ROS generation (Fig. 5f), fully inhibited Caspase-3, −8 and −9 induction (Fig. S5), and reduced apoptotic proportions in PM-exposed HBE cells compared to the control (Fig. 5g and h).

### 3.6 MT supplement attenuated pulmonary injuries in PM‑exposed mice

**Fig. 6:**
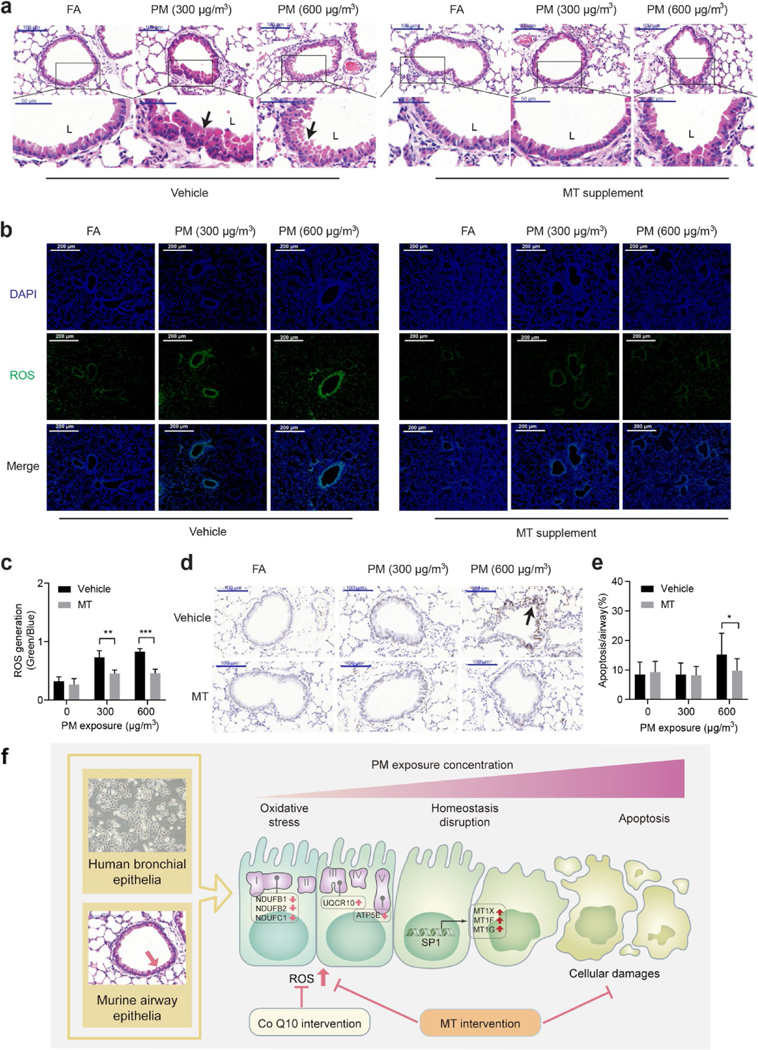
MT supplement attenuates bronchial epithelial oxidative stress and apoptosis in mice following PM exposure. **a** Representative images of small airways in mice following PM exposure by H&E staining on lung tissue sections. L: lumen of airway **b** & **c**: Representative images of ROS generation in murine lungs following PM exposure (*n* = 12/group, two-way ANOVA). **d** & **e** Representative images of TUNEL staining on lung tissue sections in mice following PM exposure (*n* = 12/group, two-way ANOVA). **f** Schematic of MT as a potential rescue against PM exposure-induced pulmonary injuries both in vitro and in vivo. * *p* < 0.05, ** *p* < 0.01, *** *p* < 0.001, compared to the PM (0 μg/mL) group

The representative image from FA/vehicle group showed normal morphology of small airway, which was composed of a single layer of cuboidal epithelia. However, in the PM-exposed murine lung, hyperplasia of the small airway epithelia was evident, which was characterized by increased layers of surface respiratory epithelial cells and desquamation into the airway lumen (L) (Fig. 6a). Images of PM/MT groups demonstrated that MT supplement effectively reversed the disruption in airway epithelium (Fig. 6a). We next determined ROS generation in murine lung tissues. As expected, increased ROS generation in airway epithelia were showed in the PM-exposed murine lungs, and MT supplement significantly inhibited ROS generation to statistically indistinguishable levels from the control (Fig. 6b and c). Finally, TUNEL assays were performed to evaluate the apoptosis in airway epithelia, which consistently corroborated ROS generation (Fig. 6d). Taken together, our study suggested MT as a potential rescue against PM exposure-induced pulmonary injuries both in vitro and in vivo (Fig. 6e).

## 4 Discussion

In the present study, we characterized the effects of PM exposure on the bronchial epithelia. Mitochondrial dysfunction, disruption in ion homeostasis, and increased oxidative stress contributed apoptosis in response to PM exposure. Notably, MT afforded anti-oxidative and anti-apoptosis efficacy, effectively rescuing bronchial epithelial cells from the damage induced by PM exposure in both mammalian cells and animals.

Cumulative evidence supports a role for oxidative stress as a critical pathway in response to PM exposure (Yue et al. 2019; Ge et al. 2020; He et al. 2017). Endogenous ROS is generated from diverse sources, including mitochondrial respiratory chain, NADPH oxidases, nitric oxide synthases, and cytochrome P450 (Nathan and Cunningham-Bussel 2013; Wende et al. 2016). Consistent with previous studies, our results demonstrated that PM exposure resulted in disturbance in the mitochondrial respiratory chains. OXPHOS dysfunction results in two major effects, namely, reduced ATP production and increased ROS production (Emma et al. 2016). In the respiratory chain, the electron carrier Co Q10 shuttles electrons from complexes I and II to complex III. Mild Co Q10 deficiency has been associated with increased ROS production, meanwhile, ATP production was not significantly changed (Quinzii et al. 2010). Co Q10 is also a well-known antioxidant and anti-apoptotic factor (Emma et al. 2016). In the present study, Co Q10 supplement partially ameliorated ROS generation, however it failed to rescue apoptosis in HBE cells following PM exposure. We thus concluded that Co Q10 acted as a general anti-oxidant, but not a specific rescue to PM exposure-induced cellular apoptosis.

Oxidative stress has been intensively associated with disruption of ion homeostasis, especially the metal ion homeostasis. Garza-Lombó et al. reviewed the role of redox signaling played in the cellular toxicity linked to xenobiotic metal exposure (Garza-Lombo et al. 2018). Though most of these studies have been conducted in the nervous system, they emphasized that disruption in homeostasis of essential metals and exposure to xenobiotic metals altered the cellular redox status and signaling. Metals are important toxic components of PM (Li et al. 2016). These metal components caused a cellular redox imbalance through cumulative formation of ROS, yet, via distinct mechanisms (Goncalves et al. 2021; Valko et al. 2016; Ying et al. 2021; Skalny et al. 2021). Therefore, over-loading of metals from PM exposure is one of the major triggers to oxidative stress in vivo. Besides, literatures also support the idea that ion homeostasis could be disturbed by PM exposure. Water-soluble ionic components of PM can easily dissolve in the wet alveolar wall and affect the alveolar cells, which directly leads to imbalanced ion homeostasis (Park et al. 2022). Air pollution, for example, may reduce sodium excretion, which enhances the risk of hypertension (Kwak and Kim 2023). The exposure of PM_2.5_ disrupts intracellular iron leading to cell ferroptosis in endothelial cells (Wang and Tang 2019). On the other hand, PM-exposure induces the aberrant expression of ion channel-related genes, which may cause disruption of ion homeostasis, leading to arrhythmia-like cardiotoxicity in zebrafish embryos (Park et al. 2023).

Previous studies have documented the contributions of several antioxidant systems to the regulation of ROS and maintenance of optimal redox status, including super-oxide dismutases, catalases and the enzymes of the glutathione redox cycle (Nathan and Cunningham-Bussel 2013). Compared with these well-known antioxidants, MT have been considered a more effective antioxidant against metal-associated oxidative stress (Carocho et al. 2018; Yu et al. 2023). The highly conserved number and position of cysteine residues inherent to MT enable them to bind physiological and xenobiotic heavy metals, as well as to reduce reactive oxygen species (Chen et al. 2024). As a stress-induced protein, MT have been reportedly involved in the underlying mechanisms to maintain metal homeostasis in cells (Witkowska et al. 2021). Corroborating to our results, it has been reported that MT are suitable candidates for exobiotic metal exposure, for their intriguing feature of high binding potential for various heavy metals, and their expressions are markedly induced during exposure to environmental insults (Mehta et al. 2016). MT in humans contains four subfamilies, among which MT1 is widely expressed in tissue to balance homeostasis. Typically, MT1G can scavenge ROS through enhancing activity of NRF2/ARE signaling without changes in free iron abundance; although MT1F and MT1X have been linked to cell proliferation, cell cycle, and apoptosis, the role of MT1F and MT1X involve in ROS was still unknown (Pavic et al. 2019; Rodrigo et al. 2020). Our results confirmed the protective role of MT-code gene, especially MT1F, in PM-exposure induced oxidative stress, ion homeostasis disruption, and apoptosis in HBE cells.

## 5 Conclusion

In summary, metal ion homeostasis and oxidative stress are major up-stream triggers of apoptosis in airway epithelia. In this context, herein, we show that induction of metal-binding antioxidants, MT, may serve as to offset and protect airway epithelia from the adverse effects of PM exposure.

## Supplementary Material

Suppl Material

## Data Availability

The mRNA microarray dataset supporting the conclusions of this article are available in the GEO repository; the accession number is GSE138870.
