# Supplementary material for: Metallothionein ameliorates airway epithelial apoptosis upon particulate matter exposure: role of oxidative stress and ion homeostasis
Source: Curr Med (Cham). Author manuscript; Available in PMC 2025 Feb 21. (PMC11845221; doi:10.1007/s44194-024-00036-7)
Supplement: Suppl Material [file NIHMS2040570-supplement-Suppl_Material.docx]

**Supplementary data for**

**Metallothionein ameliorates airway epithelial apoptosis upon particulate matter exposure: role of oxidative stress and ion homeostasis**

**Fig. S1 Expression levels of genes in HBE cells following PM exposure to SP1 KD**

a: Expression levels of SP1 in si-NC and SP1-KD HBE cells. b: Expression levels of *MT1X* and *MT1G*. c: Expression levels of *MAGOH*, *NR1I2*, *CARPRIN2*, and *SAA1*. (n=9/group, one-way ANOVA) d: Expression levels of MT1F in si-NC and MT1F-KD HBE cells. (n=9/group, *t*-test) e: Apoptosis of HBE cells (control or MT1F KD) following PM exposure. (n=3/group, two-way ANOVA) (* *p*<0.05, *** *p*<0.001)


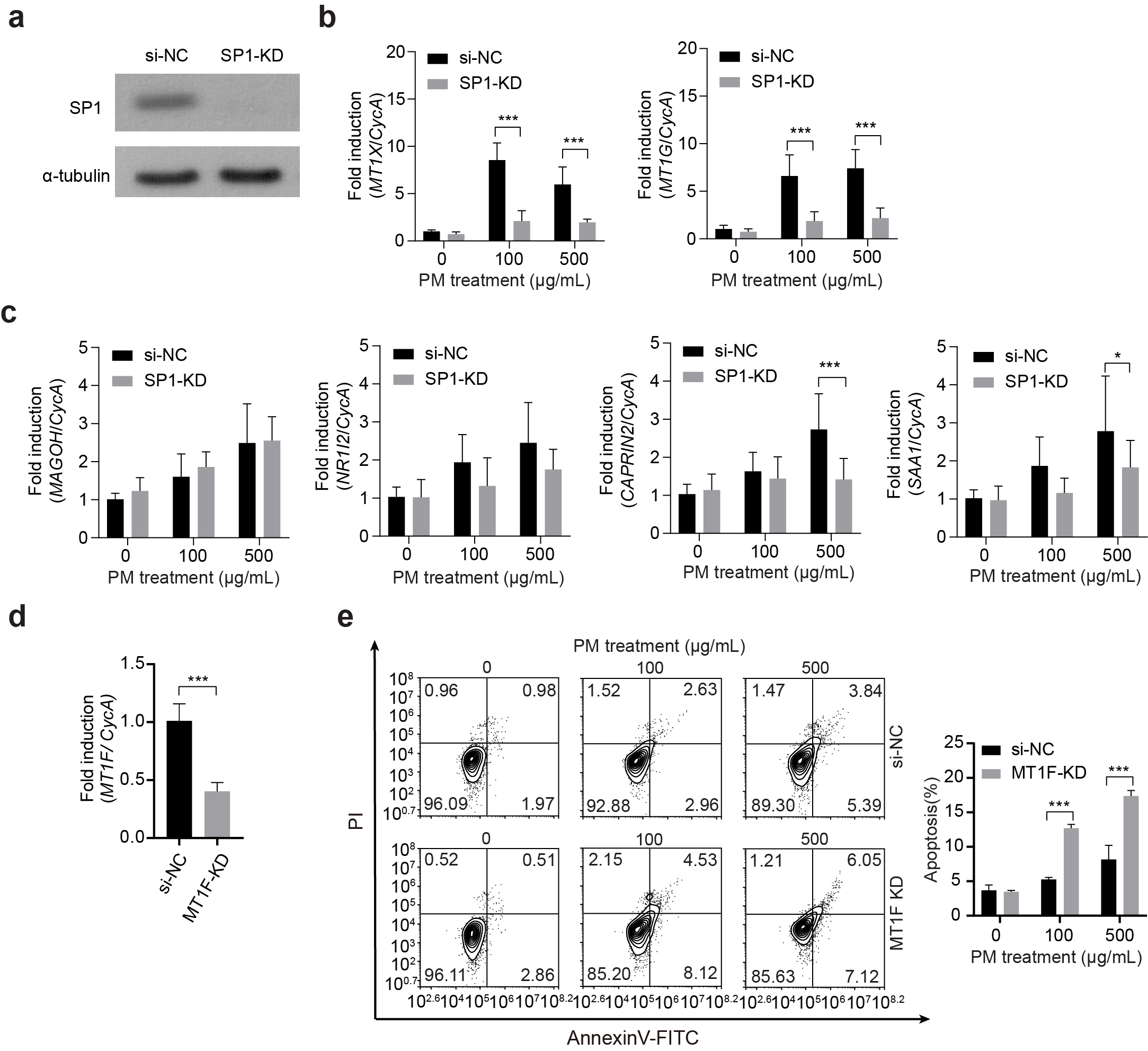


**Fig. S2 Expression levels of genes in HBE following PM exposure to Co Q10 supplement**

a: *NDUFB1*, b: *NDUFB2*, c: *NDUFC1*, d: *ATP5E* (n=9/group, one-way ANOVA)


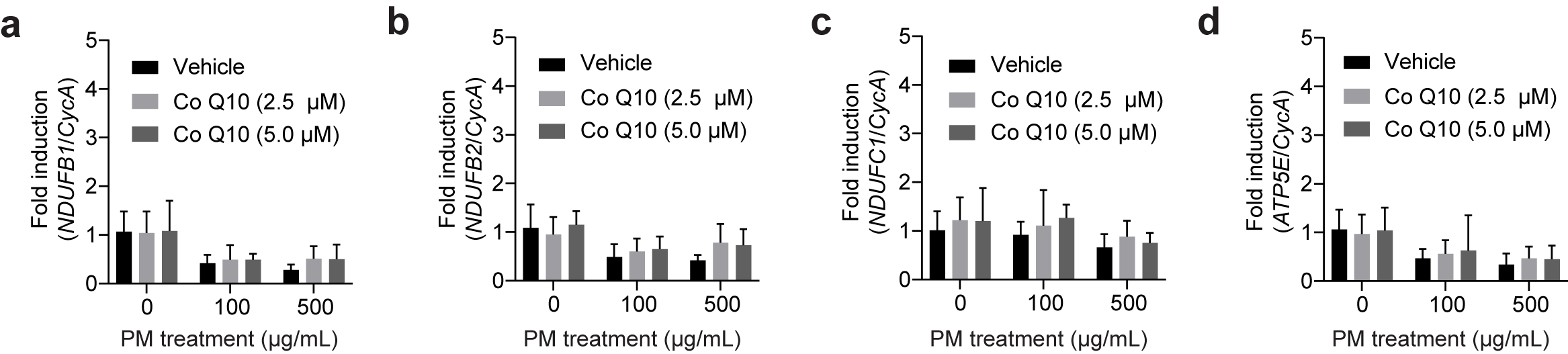


**Fig. S3 Caspase-3, -8, -9 levels in HBE cells following PM exposure to Co Q10 supplement**

a: Caspase-3 b: Caspase-8 c: Capase-9. (n=3/group, two-way ANOVA, * *p*<0.05)

**
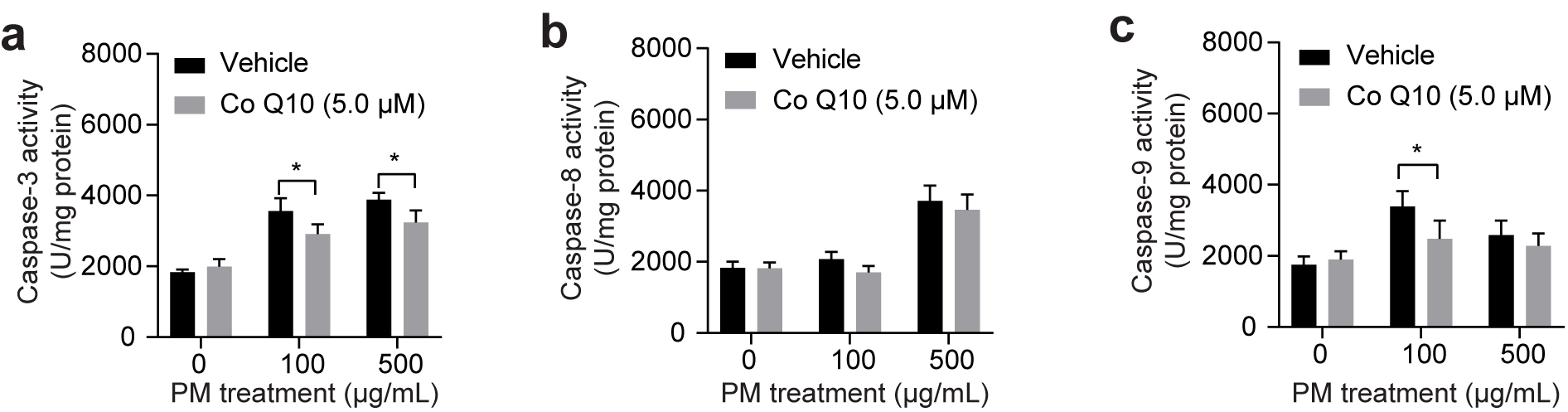
**

**Fig. S4 Expression levels of genes in HBE cells following PM exposure with MT supplement**

a: *MT1F*, b: *NDUFC1*, c: *NDUFB1*, d: *NDUFB2*, e: *UQCR10*, f: *ATP5E* (n=9/group, one-way ANOVA, ** *p*<0.01, *** *p*<0.001)


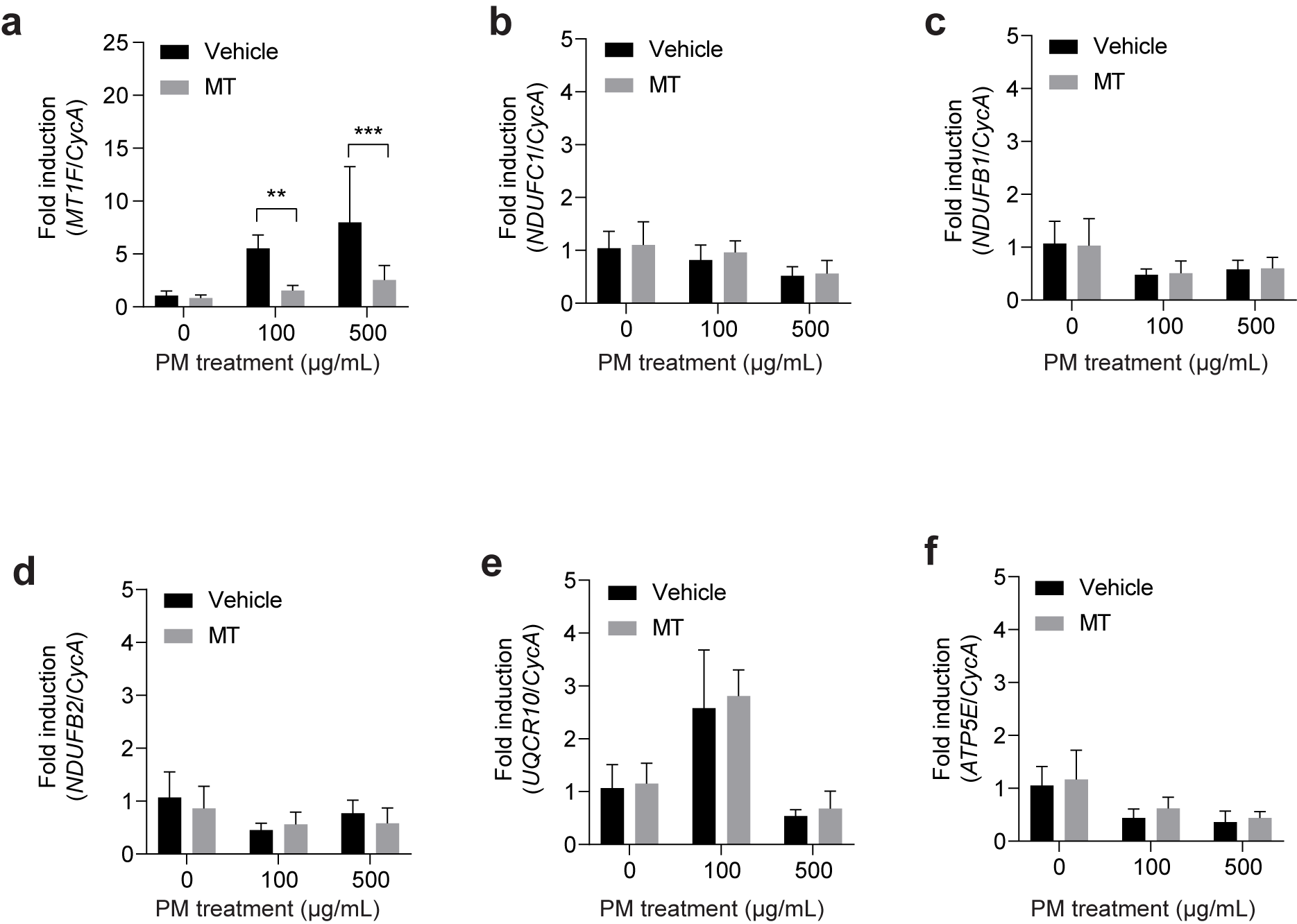


**Fig. S5 Caspase-3, -8, -9 levels in HBE cells following PM exposure with MT supplement**

a: Caspase-3 b: Caspase-8 c: Capase-9. (n=3/group, two-way ANOVA, ** *p*<0.01, *** *p*<0.001)

**
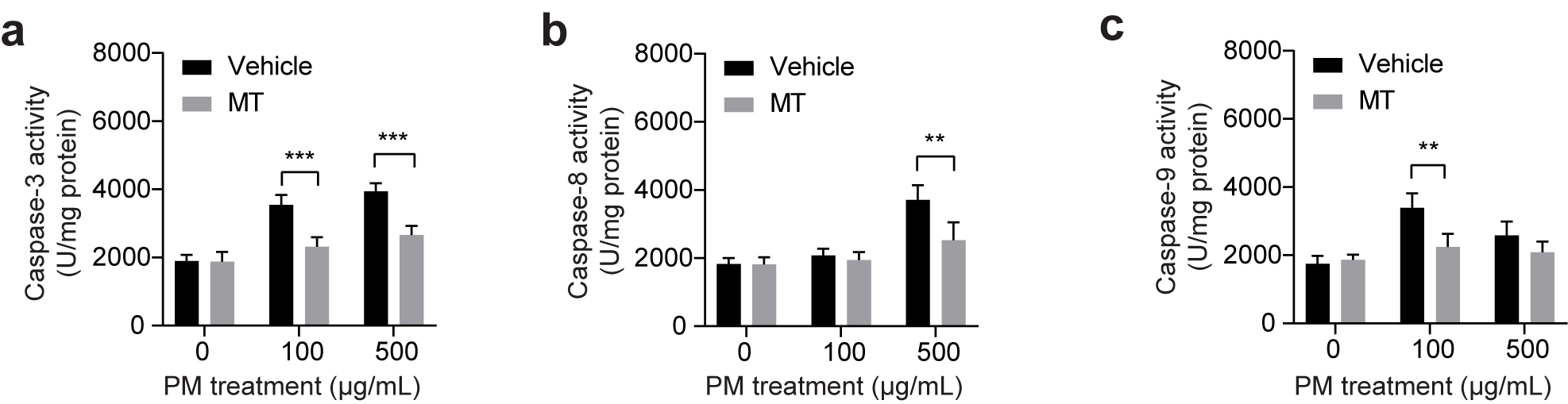
**

**Table S1 Primer sequences for RT-PCR assay**

|  | Forward | Reverse |
| --- | --- | --- |
| *ATP5E* | 5’-GGCTGGACTCAGCTACATCC-3’ | 5’-TTACGTTGCTGCCAGAAGTCT-3’ |
| *NDUFC1* | 5’-CCTTCAGTGCGATCAAAGTTCT-3’ | 5’-CAGCCAGTCAGGTTTGGCAT-3’ |
| *NDUFB1* | 5’-GTCCCTATGGGATTTGTCATTGG-3’ | 5’-CAGTTAGCCGTTCATCACTCTT-3’ |
| *NDUFB2* | 5’-GGAGGCCGCCTTTTCAGAA-3’ | 5’-GGAAGGATCAGGATACGGAAAGT-3’ |
| *UQCR10* | 5’-ATCGTGGGCGTCATGTTCTTC-3’ | 5’-ATGTGGTCGTAGATAGCGTCC-3’ |
| *CD38* | 5’-CAACTCTGTCTTGGCGTCAGT-3’ | 5’-CCCATACACTTTGGCAGTCTACA-3’ |
| *CASP5* | 5’-TTCAACACCACATAACGTGTCC-3’ | 5’-GTCAAGGTTGCTCGTTCTATGG-3’ |
| *BIRC5* | 5’-AGGACCACCGCATCTCTACAT-3’ | 5’-AAGTCTGGCTCGTTCTCAGTG-3’ |
| *UNC5B* | 5’-GTCGGACACTGCCAACTATAC-3’ | 5’-CCGCCATTCACGTAGACGAT-3’ |
| *MEF2C* | 5’-CCAACTTCGAGATGCCAGTCT-3’ | 5’-GTCGATGTGTTACACCAGGAG-3’ |
| *SERPINB9* | 5’-AATGCAAGTGGTACTTTTGCCA-3’ | 5’-AAGCCCGATGAATGTCTTCCT-3’ |
| *JUN* | 5’-TCCAAGTGCCGAAAAAGGAAG-3’ | 5’-CGAGTTCTGAGCTTTCAAGGT-3’ |
| *NRAS* | 5’-ATGACTGAGTACAAACTGGTGGT-3’ | 5’-CATGTATTGGTCTCTCATGGCAC-3’ |
| *GDF5* | 5’-GCTGGGAGGTGTTCGACATC-3’ | 5’-CACGGTCTTATCGTCCTGGC-3’ |
| *MAGOH* | 5’-TTTATCTGCGTTACTACGTGGGG-3’ | 5’-CGTCCGGTCGAAACTCAAACT-3’ |
| *CAPRIN2* | 5’-TCTCAAGCATCATTGGGTTTCG-3’ | 5’-CAGCCAGGCAATAACTTCCCT-3’ |
| *NR1I2* | 5’-TTGCCCATCGAGGACCAGAT-3’ | 5’-GTCTCCGCGTTGAACACTGT-3’ |
| *MT1X* | 5’-CTCCTTGCCTCGAAATGGAC-3’ | 5’-AGCTGCACTTGTCTGACG-3’ |
| *MT1G* | 5’-CATCTGCAAAGGGGCATC-3’ | 5’-CTGGATTTTACGGGTCACTC-3’ |
| *MT1F* | 5’-ACTGCTTCTTCGCTTCTC-3’ | 5’-CAAATGGGTCAAGGTGGTA-3’ |
| *SAA1* | 5’-GCTTCTTTTCGTTCCTTGGCG-3’ | 5’-GCCGATGTAATTGGCTTCTCTCA-3’ |

**Table S2 The primer sequences of human genes for ChIP assay**

|  | Forward | Reverse |
| --- | --- | --- |
| *MT1X* | 5’-CCATTCAGCCTCTCAAGA-3’ | 5’-GGATGTGACACTCGGATT-3’ |
| *MT1G* | 5’-ACCTTGCACTTGGCCCAT-3’ | 5’-GCCCCGCCCTTGCACC-3’ |
| *MT1F* | 5’-GGTGTAGCAGGCAATCTT -3’ | 5’-CTTTGGAGTCCGCTCAAG-3’ |

**Table S3 Target sequences of siRNAs**

| siRNAs |  | Target Sequence |
| --- | --- | --- |
| *si-SP1* |  | 5’-GCTGGTGGTGATGGAATACAT-3’ |
| *si-MT1F* |  | 5’-GCAAGTGCAAAGAGTGCAAAT-3’ |

**Table S4 BP terms of differentially regulated genes in HBE cells following 100 μg/ml PM exposure**

| BP term | *p*-value | Genes |
| --- | --- | --- |
| Oxidative phosphorylation | 0.0014 | *ATP5E, UQCR10, NDUFC1, NDUFB1, NDUFB2* |
| ATP synthesis coupled electron transport | 0.0027 | *UQCR10, NDUFC1, NDUFB1, NDUFB2* |
| Mitochondrial ATP synthesis coupled electron transport | 0.0027 | *UQCR10, NDUFC1, NDUFB1, NDUFB2* |
| Defense response | 0.0035 | *ABCF1, NLRP7, REG3A, HIST2H2BE, SAA1, GAGE2A, MSH5, TLR3, MIF, CLEC1B* |
| Respiratory electron transport chain | 0.0040 | *UQCR10, NDUFC1, NDUFB1, NDUFB2* |
| Di-, tri-valent inorganic cation homeostasis | 0.0069 | *CCL1, MCHR1, SAA1, CCDC47, CHRFAM7A, MON1A* |
| Cellular respiration | 0.0127 | *UQCR10, NDUFC1, NDUFB1, NDUFB2* |
| Cation homeostasis | 0.0143 | *CCL1, MCHR1, SAA1, CCDC47, CHRFAM7A, MON1A* |
| Calcium ion homeostasis | 0.0147 | *CCL1, MCHR1, SAA1, CCDC47, CHRFAM7A* |
| Mitochondrial electron transport, NADH to ubiquinone | 0.0188 | *NDUFC1, NDUFB1, NDUFB2* |
| Metal ion homeostasis | 0.0196 | *CCL1, MCHR1, SAA1, CCDC47, CHRFAM7A* |
| Electron transport chain | 0.0196 | *UQCR10, NDUFC1, NDUFB1, NDUFB2* |
| Generation of precursor metabolites and energy | 0.0204 | *ATP5E, MCHR1, UQCR10, NDUFC1, NDUFB1, NDUFB2* |
| Cellular macromolecular complex assembly | 0.0217 | *PIH1D1, HIST1H1C, HIST2H2BE, SNRPD2, CELF1, TUBA1C* |
| Cellular di-, tri-valent inorganic cation homeostasis | 0.0272 | *CCL1, MCHR1, SAA1, CHRFAM7A, MON1A* |

**Table S5 BP terms of differentially regulated genes in HBE cells following 500 μg/ml PM exposure**

| BP term | *p*-value | Genes |
| --- | --- | --- |
| Regulation of apoptosis | 0.0169 | *MEF2C, CASP5, NRAS, CD38, SERPINB9, JUN, GDF5, BIRC5, BIRC3* |
| Regulation of programmed cell death | 0.0179 | *MEF2C, CASP5, NRAS, CD38, SERPINB9, JUN, GDF5, BIRC5, BIRC3* |
| Regulation of cell death | 0.0182 | *MEF2C, CASP5, NRAS, CD38, SERPINB9, JUN, GDF5, BIRC5, BIRC3* |
| Cellular ion homeostasis | 0.0189 | *CD38, SAA1, JUN, MT2A, CHRFAM7A, MT3* |
| Male gonad development | 0.0195 | *NR0B1, ANKRD7, MGST1* |
| Cellular chemical homeostasis | 0.0201 | *CD38, SAA1, JUN, MT2A, CHRFAM7A, MT3* |
| Cellular cation homeostasis | 0.0207 | *CD38, SAA1, MT2A, CHRFAM7A, MT3* |
| Ion homeostasis | 0.0266 | *CD38, SAA1, JUN, MT2A, CHRFAM7A, MT3* |
| Leydig cell differentiation | 0.0286 | *NR0B1, MGST1* |
| Development of primary male sexual characteristics | 0.0296 | *NR0B1, ANKRD7, MGST1* |
| Cation homeostasis | 0.0304 | *CD38, SAA1, MT2A, CHRFAM7A, MT3* |
| Male sex differentiation | 0.0366 | *NR0B1, ANKRD7, MGST1* |
| Apoptosis | 0.0374 | *MEF2C, CASP5, NRAS, UNC5B, JUN, BIRC5, BIRC3* |
| Programmed cell death | 0.0397 | *MEF2C, CASP5, NRAS, UNC5B, JUN, BIRC5, BIRC3* |
| Cellular homeostasis | 0.0430 | *CD38, SAA1, JUN, MT2A, CHRFAM7A, MT3* |
| Cellular metal ion homeostasis | 0.0474 | *CD38, SAA1, CHRFAM7A, MT3* |

**Table S6 BP terms of overlapped differentially regulated genes in HBE cells following 100 or 500 μg/ml PM exposure**

| BP Term | *p*-value | Genes |
| --- | --- | --- |
| Di-, tri-valent inorganic cation homeostasis | 0.0010 | *CCL1, MCHR1, SAA1, CCDC47, CHRFAM7A, MON1A* |
| Cation homeostasis | 0.0022 | *CCL1, MCHR1, SAA1, CCDC47, CHRFAM7A, MON1A* |
| Calcium ion homeostasis | 0.0031 | *CCL1, MCHR1, SAA1, CCDC47, CHRFAM7A* |
| Metal ion homeostasis | 0.0043 | *CCL1, MCHR1, SAA1, CCDC47, CHRFAM7A* |
| Cellular di-, tri-valent inorganic cation homeostasis | 0.0061 | *CCL1, MCHR1, SAA1, CHRFAM7A, MON1A* |
| Cellular cation homeostasis | 0.0091 | *CCL1, MCHR1, SAA1, CHRFAM7A, MON1A* |
| Ion homeostasis | 0.0101 | *CCL1, MCHR1, SAA1, CCDC47, CHRFAM7A, MON1A* |
| Defense response | 0.0139 | *ABCF1, NLRP7, REG3A, SAA1, MSH5, TLR3, CLEC1B* |
| Cellular calcium ion homeostasis | 0.0214 | *CCL1, MCHR1, SAA1, CHRFAM7A* |
| Chemical homeostasis | 0.0245 | *CCL1, MCHR1, SAA1, CCDC47, CHRFAM7A, MON1A* |
| Cellular metal ion homeostasis | 0.0256 | *CCL1, MCHR1, SAA1, CHRFAM7A* |
| Cellular ion homeostasis | 0.0327 | *CCL1, MCHR1, SAA1, CHRFAM7A, MON1A* |
| Negative regulation of cell differentiation | 0.0329 | *HDAC1, NME1, TLR3, NR0B1* |
| Cellular chemical homeostasis | 0.0344 | *CCL1, MCHR1, SAA1, CHRFAM7A, MON1A* |
| Negative regulation of myeloid leukocyte differentiation | 0.0539 | *NME1, TLR3* |
